# Tuberculosis and Impact of COVID-19 on Spread of Epidemics in Kazakhstan

**DOI:** 10.3390/pathogens14060559

**Published:** 2025-06-04

**Authors:** Zhandarbek Bekshin, Albert Askarov, Yergali Abduraimov, Aralbek Rsaliyev, Gulmira Bissenova, Nurgul Amirkhanova, Zhadyrassyn Nurbekova, Aliya Temirbekova

**Affiliations:** 1 Republican Collection of Microorganisms, Astana 010000, Kazakhstan; 2 National Center for Biotechnology, Astana 010000, Kazakhstan; 3 National Holding Qazbiopharm, Astana 010000, Kazakhstana.rsaliyev@qbp-holding.kz (A.R.); 4 Department of Biotechnology and Microbiology, L.N. Gumilyov Eurasian National University, Astana 010000, Kazakhstan

**Keywords:** tuberculosis, *Mycobacterium tuberculosis*, TB incidence, TB mortality, vaccination, unemployment, epidemiological situation, infectious diseases

## Abstract

This study examines the epidemiological situation of tuberculosis (TB) in the regions of the Republic of Kazakhstan over the past seven years (2018–2024), which cover the before-, during- and after-COVID-19 periods, with a focus on the risks of its emergence and spread. The analysis revealed that while TB incidence is declining, mortality remains high in the before- and during-COVID-19 periods, indicating a general decline in population health. The concentration of TB incidence in relation to geographic location was mainly in the northern, western and southern regions. Before COVID-19, TB incidence reached 48.2 cases and mortality reached a maximum of 2.4 cases per 100,000 people. In 2024, the incidence and mortality of tuberculosis significantly decreased to 33.5 (30.5%) and 1.0 (58.3%), respectively, reflecting an improvement in health indicators in the post-pandemic period. In the after-COVID-19 period, in regions with high unemployment, the incidence was higher than in the before- and during-COVID-19 periods. Nevertheless, it is important that the trend in tuberculosis incidence shows positive improvement after the COVID-19 period. In addition, a comparative analysis of tuberculosis incidence trends in different age groups and social factor groups shows that the adult population remains the most vulnerable category among the general population. The above-listed factors, as well as our analysis of tuberculosis incidence, shows that TB incidence does not always correlate with the level of vaccination in different regions of Kazakhstan, indicating a multifactorial influence on the tuberculosis epidemic.

## 1. Introduction

Tuberculosis (TB) is one of the oldest airborne diseases caused by *Mycobacterium tu-berculosis* bacteria [1]. TB is mainly transmitted by airborne and droplet transmission from individuals with the infection [2,3,4]. Droplet infections are transmitted by coughing and play a leading role in the spread of TB [2,5,6].

This pathogen most commonly affects the lungs, resulting in severe respiratory manifestations with persistent cough, fever, chest pain and, in some cases, even death [7,8]. Tuberculosis remains a global and significant public health problem, causing approximately 2 million deaths and infecting nearly 9 million people worldwide each year [9]. It is reported that the World Health Organization (WHO) estimated 10.4 million new cases of tuberculosis in 2015 [10].

In 2021, the Southeast Asia region had the highest number of new TB cases, accounting for 46% of all cases globally and 50% of all TB-related deaths. This was followed by the Africa region, with 23% of all TB cases globally, while the western Pacific region had 18% [11,12]. Kazakhstan is also facing a challenging situation regarding the incidence of tuberculosis in its population [13].

Despite advances in prevention, treatment and control strategies that have helped bring tuberculosis under control, it remains a major global threat, ranking second after HIV/AIDS in terms of mortality in the 21st century [14]. There are several key challenges in controlling tuberculosis such as antibiotic resistance, limited public awareness, limited access to diagnostic tools and inadequate health facilities [15]. Mycobacteria are classified as acid-fast bacilli with a unique cell wall structure containing glycolipids that also contribute to their resistance to antibiotics and host defense mechanisms [16,17].

In order to control the epidemiologic situation of tuberculosis, it is crucial to study the risks associated with the main routes of transmission: aerogenic, contact and other vectors [9,10]. The prevention and control of epidemiologic diseases require a multidisciplinary approach involving public health, veterinary and environmental experts [13,14]. Critical measures include good hygiene practices, animal vaccination and the surveillance and early detection of tuberculosis cases [18].

Various social and demographic factors, such as alcohol dependence, low income, immigration, co-infections and poor living conditions including overcrowding, unregulated heat, limited ventilation and malnutrition, are known to influence TB transmission [19]. Each of these elements contributes to impaired immune response and increased exposure, creating an environment in which tuberculosis transmission can increase significantly [20]. It has been reported that the COVID-19 pandemic has had an impact on the spread of tuberculosis worldwide, including in Kazakhstan [7]. Kazakhstan is one of the thirty countries with multidrug-resistant TB according to the WHO. However, comparative studies of TB incidence before, after and during COVID-19 remain largely inconclusive.

Therefore, in this study, we examined the prevalence of TB before, during and after COVID-19 in Kazakhstan, including other factors such as social and geographic determinants in different regions throughout the country.

## 2. Methods

### 2.1. Study Area and Period

This study aims to investigate the epidemiologic situation of tuberculosis in the Republic of Kazakhstan in the period from 2018 to 2024.

### 2.2. Data Collection

Data on tuberculosis (TB) incidence and mortality were taken from the annual republican reports of the Ministry of Health of the Republic of Kazakhstan [21]. In the country, with an area of 2724.9 thousand square kilometers, 17 regions (14 in 2018; 3 new regions were created in 2022—Abai, Zhetisu and Ulytau) and 3 megacities (Almaty, Astana and Shymkent) were identified. We analyzed data on TB incidence over a seven-year period (2018–2024) across the country, focusing on three main age groups: children aged 0–14 years, adolescents aged 15–17 years and adults aged 18 years and above.

### 2.3. Data Analysis

This study used retrospective, cartographic and statistical methods of analysis to summarize data on the epidemiological situation of tuberculosis in Kazakhstan, social factors and pro-preventive measures. These approaches were used to determine the frequency and distribution of diseases (outcomes) in the territory of Kazakhstan (cities) for certain periods of time (seven years). Tuberculosis incidence was quantified as the number of patients diagnosed with active tuberculosis, and tuberculosis mortality was quantified as the number of tuberculosis deaths for the whole year in a certain territory. The incidence rate is calculated as follows: the number of TB cases in a certain territory (of all ages) per year is multiplied by 100,000 and divided by the population of that territory per year (according to the Statistics Committee of the Republic of Kazakhstan). Similarly, the rates are calculated by region or age (0–14 years old, adolescents 15–17 years old, adults 18 years and older). This is the indicator of incidence per 100 thousand population. The mortality rate is calculated in the same manner: the number of TB deaths is multiplied by 100 thousand population (10,000, etc.) and divided by the population of the republic, region, etc.

Temporal and regional trends in TB incidence and mortality were assessed and visualized using geographic information system (GIS) tools, using the QGIS interface to create spatial maps of TB incidence and mortality distributions. To study the regional dynamics of incidence and mortality, five levels were distinguished according to the indicators per 100,000 people relative to all periods, which include before, during and after COVID-19—based on the highest incidence rate of 76.4 and the highest mortality rate of 4.5 for the entire seven-year period, 5 equal ranges were identified in both cases:

- TB incidence ranges defined as follows: 0.00–44.0—low level of incidence; 44.0–47.5—slightly below average; 47.5–51.8—average and slightly above average; 51.8–55.4—high; and 55.4–74.8—very high.
- TB mortality ranges: 0.0–1.48—low; 1.48–2.2—below average; 2.2–2.7—average and slightly above average; 2.7–2.8—high; and 2.8–4.5—very high.

Visualization of vaccination rate data regarding the dynamics of TB incidence was performed in the Jupyter Lab 3.6 interactive environment, using Python 3.11 libraries (numpy, matplotlib).

In addition, this study analyzed the seven-year dynamics of TB incidence both at the population level and stratified by socioeconomic status and age groups. Unemployment and regional urbanization data were obtained from the Bureau of National Statistics under the Agency for Strategic Planning and Reforms of the Republic of Kazakhstan [22].

### 2.4. Statistical Analysis

Statistical analyses were carried out by using GraphPad Prism software, version 10. TB incidence and TB mortality data during the COVID-19 pandemic period were analyzed by a One-way ANOVA test and Tukey test. The effect of the regional and socioeconomic factors on TB incidence were analyzed by using the multiple linear regression (MLR) test. All statistical analyses were performed according to the *p* value, with *p* < 0.05.

### 2.5. Ethical Considerations

No human research was conducted in this paper, so it does not require ethical approval.

## 3. Results

**Figure 1:**
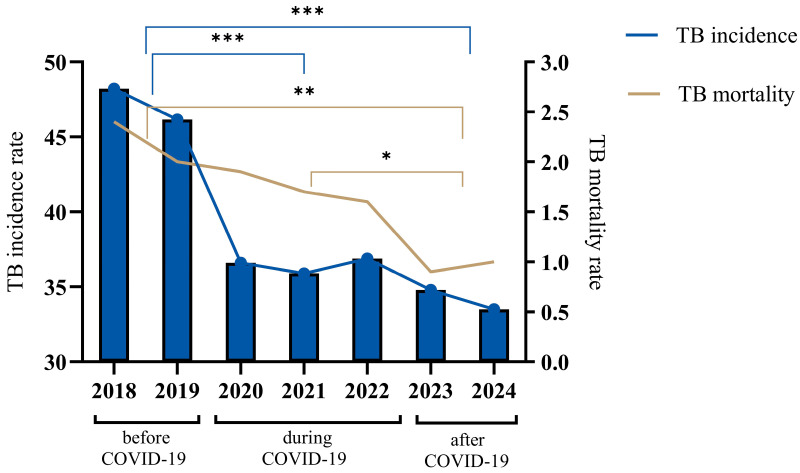
Tuberculosis incidence and mortality trends for the years 2018–2024. The figure illustrates the annual TB incidence (cases per 100,000 population) and mortality rates (deaths per 100,000 population) across three distinct periods: before COVID-19 (2018–2019), during COVID-19 (2020–2021) and after COVID-19 (2022–2024). TB incidence is represented by vertical bars with connecting blue lines, showing a significant decline over the observed years. TB mortality rates, depicted by the continuous brown line, similarly demonstrate a steady decrease throughout the entire period, indicating improvement in TB management and control measures during and after the COVID-19 pandemic. The data were analyzed by a One-way ANOVA test with a Tukey test, and the *p* value indicates a significant difference. The asterisks (*, **, ***) represent levels of statistical significance for differences in TB incidence rates between population groups and across years, where * indicates significant, ** indicates highly significant, *** indicates very highly significant differences.

During this study, a comparative analysis of the dynamics of incidence and mortality for tuberculosis (TB) in Kazakhstan during different time periods of COVID-19 was carried out (Figure 1). Before the COVID-19 period (2018–2019), there was a gradual decrease in tuberculosis incidence, while the disease incidence rate (per 100,000 population) in the country in 2019 compared to 2018 decreased by 4.17% and amounted to 46.2 compared to 48.2 in 2018. The gradual decrease in TB incidence during the 2018–2019 period was replaced by a sharp decline at the beginning of COVID-19, and the TB incidence rate in 2020 decreased to 36.6, which is 20.7% lower as compared to 2019. Then, in the middle of the COVID-19 period, the incidence rate decreased to 35 in 2021, which is a 0.6% decrease. However, at the end of the COVID-19 period, the incidence rate increased to 36.9 in 2022, which is a 2.7% increase. After the COVID-19 period (2023), the TB incidence rate in the population decreased significantly to 34.8 in 2022, which is a 5.6% decrease. The TB incidence rate of the population in the republic for 2024 decreased to 33, which is a 3.4% decrease.

Regarding the tuberculosis mortality rate (per 100 thousand people), starting in the before-COVID-19 period (2018), there was a gradual decline until the end of the COVID-19 period (2022), from an indicator value of 2.4 to 1.6, which is a 33.3% decrease. In 2023, the mortality rate decreased to 1.2, but in 2024, the mortality rate increased to 1.0, an increase of 16.7%.

In general, over the seven-year period, from 2018 to 2024, Kazakhstan achieved a notable decrease in TB incidence, with the incidence rate declining by 30.5%, from 48.2 to 33.5 per 100,000 people, and mortality by 58.3%, from 2.4 to 1.0 per 100,000 people.

**Figure 2:**
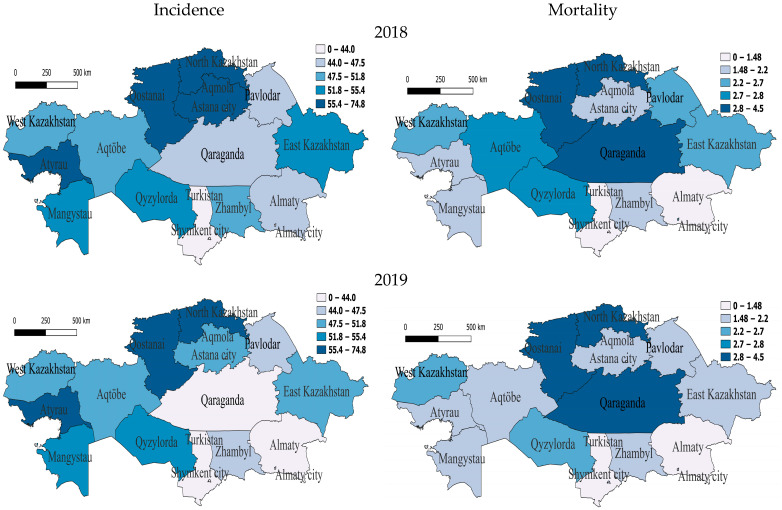
Geographic distribution of tuberculosis (TB) incidence and mortality rates in Kazakhstan before COVID-19 for the years 2018 and 2019. Left-side maps depict the regional variation in TB incidence rates (cases per 100,000 population), whereas right-side maps illustrate corresponding mortality rates (deaths per 100,000 population). Upper panels represent data from 2018, while lower panels illustrate the situation in 2019. Darker shading indicates higher incidence or mortality rates, highlighting regional disparities and temporal changes in TB epidemiology across Kazakhstan.

We analyzed the regional dynamics of TB incidence and mortality rates per 100,000 people in relation to their numerical scales derived from the indicators for the entire seven-year period. Before the COVID-19 period, in 2018, in the western region of Atyrau and in three northern regions—North Kazakhstan, Qostanai and Akmola—the rates were between 55.4 and 74.8, indicating a very high incidence rate. Furthermore, two western regions—Mangistau and Aqtobe—one southern region—Qyzylorda—and one eastern region—East Kazakhstan—were classified as having high morbidity rates, as their rates were between 51.8 and 55.4. The southern region Zhambyl, the northern region Astana and the western region West Kazakhstan were characterized by slightly above-average incidence rates, while the rest were classified as having slightly below-average or lower incidence rates. High mortality at this time was observed in North Kazakhstan, Qostanai and Qaraganda (Figure 2).

In 2019, regions with very high incidence also included one western region—Atyrau—and two northern regions—North Kazakhstan and Qostanai. The regions with high incidence were one south-western region—Mangistau—and one southern region—Qyzylorda. Those with slightly above-average incidence were two western regions—Aqtobe and West Kazakhstan—one northern region—Aqmola—and one eastern region—East Kazakhstan. In the two northern regions of Astana and Pavlodar and one southern region, Zhambyl, the incidence rate was slightly below average for the whole seven-year period but, relative to the national indicator, remained slightly higher, which still indicates a decrease in the incidence rate in this region (Figure 2). In 2019, there was high mortality in two northern regions—North Kazakhstan and Qostanai—and one central region—Qaraganda. At the same time, mortality in one western region—Aqtobe—and in East Kazakhstan decreased by 25% and 25.9% to below average, and the rest had low mortality (Figure 2).

**Figure 3:**
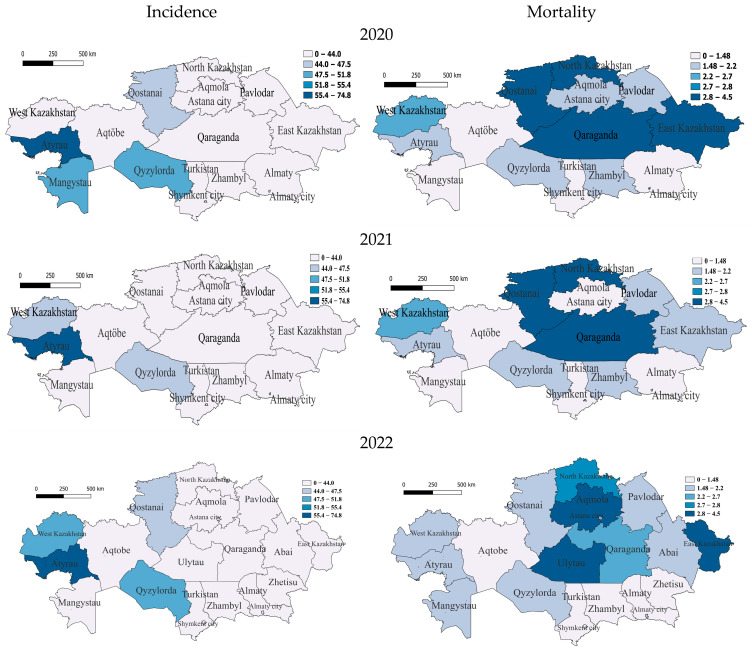
Geographic distribution of tuberculosis (TB) incidence and mortality rates in Kazakhstan during COVID-19 for the years 2020–2022. Left-side maps depict the regional variation in TB incidence rates (cases per 100,000 population), whereas right-side maps illustrate corresponding mortality rates (deaths per 100,000 population). The top maps correspond to data from 2020, the middle maps represent 2021, and the bottom maps illustrate the situation in 2022. Darker shading indicates higher incidence or mortality rates, highlighting spatial differences and temporal trends across Kazakhstan during the COVID-19 pandemic period.

At the beginning of the COVID period, in 2020, the only region with a very high incidence rate was, again, in the west, Atyrau (56.9). It is noteworthy that the Atyrau region was characterized by a very high incidence rate throughout the before- and during-COVID-19 periods. The regions with medium incidence were the southern region of Qyzylorda, the south-western region of Mangistau and the northern region of Qostanai, with the rest having low incidence. For this year, the group of regions with very high mortality from tuberculosis included two northern regions, North Kazakhstan and Qostanai, one in the east—East Kazakhstan—and one in the center—Qaraganda. The regions with average and slightly above-average mortality were Almaty (megacity) in the south and two in the west—West Kazakhstan and Atyrau—with the rest having average and below-average mortality (Figure 3).

In 2021, the only region with very high incidence remains Atyrau. Further regions with incidence slightly below average are the southern region of Qyzylorda and West Kazakhstan, with the rest having low incidence, which indicates a significant improvement in the epidemiological situation in the country. High TB mortality rates in 2021 remained in the north—in North Kazakhstan and Qostanai—and in the center—Qaraganda. Regions with average and slightly below-average mortality rates were Atyrau, West Kazakhstan and East Kazakhstan, and the rest had low mortality rates (Figure 3).

In 2022, at the end of the COVID period, the Atyrau (59.6) region also belongs to the most vulnerable category, but the incidence is gradually decreasing. Only the following regions belong to the category with average incidence: West Kazakhstan in the west and Qyzylorda in the south, respectively. One northern region—Qostanai—belongs to the category with slightly below-average morbidity, with the rest having low morbidity. In terms of mortality, the regions of Ulytau (in the center), East Kazakhstan (in the east) and Aqmola (in the north) remained at a very high level. North Kazakhstan had high mortality, and one more region, Qaraganda, was classified as a medium-level region, with the rest having below-average and low mortality (Figure 3).

**Figure 4:**
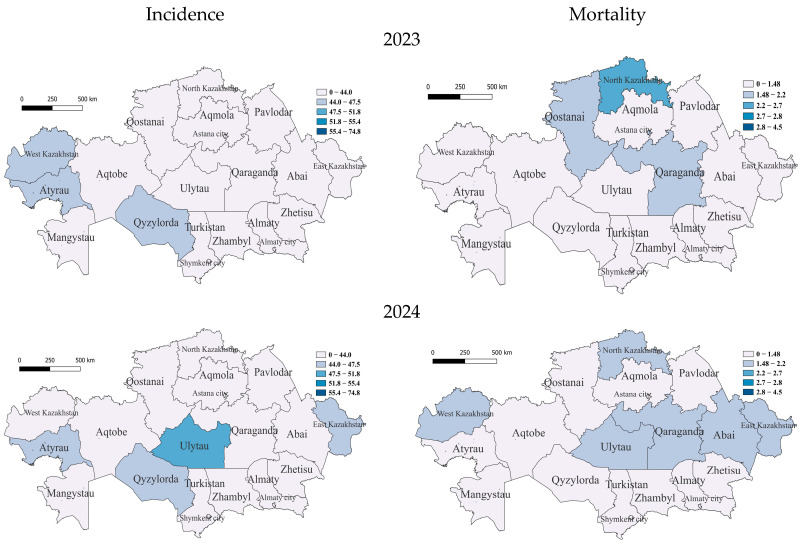
Geographic distribution of tuberculosis (TB) incidence and mortality rates in Kazakhstan after COVID-19 for the years 2023 and 2024. Left-side maps show TB incidence rates (cases per 100,000 population), and right-side maps illustrate mortality rates (deaths per 100,000 population). Upper panels correspond to 2023 data, and lower panels represent the year 2024. The gradual shift toward lighter shading (white color) in these maps indicates significant improvements in the regional TB situation, reflecting reduced incidence and mortality rates across Kazakhstan.

In the COVID-19 period, in 2023, only three regions belonged to the group with incidence rates slightly below average—the southern region of Qyzylorda and Western Kazakhstan and Atyrau, already included in this group, in the west, with the rest of the regions having relatively low incidence rates. With an average mortality rate in 2023 was the region of North Kazakhstan; furthermore, those with below-average mortality rates were the regions of Qaraganda, East Kazakhstan, Qostanai and Astana, with the rest having low rates (Figure 4).

In 2024, unexpectedly, in the central region of Ulytau, the incidence reached a high level. East Kazakhstan, Atyrau and Qyzylorda became regions with below-average incidence rates, while the rest of the regions were classified as low. There were high TB mortality rates in Qaraganda, West Kazakhstan, Ulytau, East Kazakhstan, Abai and North Kazakhstan (Figure 4).

Furthermore, to identify the possible influence of such factors as regional factors, unemployment rate and urbanization level on the incidence rate (source—Bureau of National Statistics of the Agency for Strategic Planning and Reforms of the Republic of Kazakhstan), multiple linear regression analysis was used (megalopolises such as Astana city, Almaty city and Shymkent city were excluded from this analysis, as they are capable of distorting the regression estimate due to extreme values of urbanization—100%) (Table 1). The form of the model is as follows:

A ~ Intercept + β*i* B + β*i* C + β*i* D (1)

where A is the dependent variable (TB incidence rate); B, C and D are independent variables (B—unemployment rate; C—urbanization rate; D—region); and β*i* (*i* = 0, 1, ⋯) is the regression coefficient of the independent variable. The analysis covered the entire study period, from 2018 to 2024.

According to the results of multiple linear regression analysis, unemployment and urbanization factors had an inverse effect on TB incidence. With an increase in the unemployment rate, the TB incidence rate tended to decrease, which indicates the absence of its influence, where it did not have a significant probability. On the other hand, with an increase in the level of urbanization, TB incidence also tended to decrease, which indicates its positive effect. In addition, the regional factor had a strong effect on TB incidence. For example, the Turkistan region (reference, since it had the lowest incidence rate), Almaty region, Zhetysu region and Zhambyl region corresponded to a decrease in TB incidence rates, and regions such as Qaraganda, Ulytau, Aqtobe, Pavlodar and Atyrau, on the contrary, corresponded to an increase in TB incidence. The West Kazakhstan region and the Qyzylorda region did not have an impact on the incidence rate (*p* value < 0.05). The asterisks (**, ****) represent levels of statistical significance for differences in TB incidence rates between population groups and across years, where ** indicates highly significant and **** indicates extremely significant differences.

### Vaccination Effects on TB Incidence Rates

One of the main preventive measures for tuberculosis among children is the preventive vaccination of newborns in the conditions of maternity hospitals or wards.

**Figure 5:**
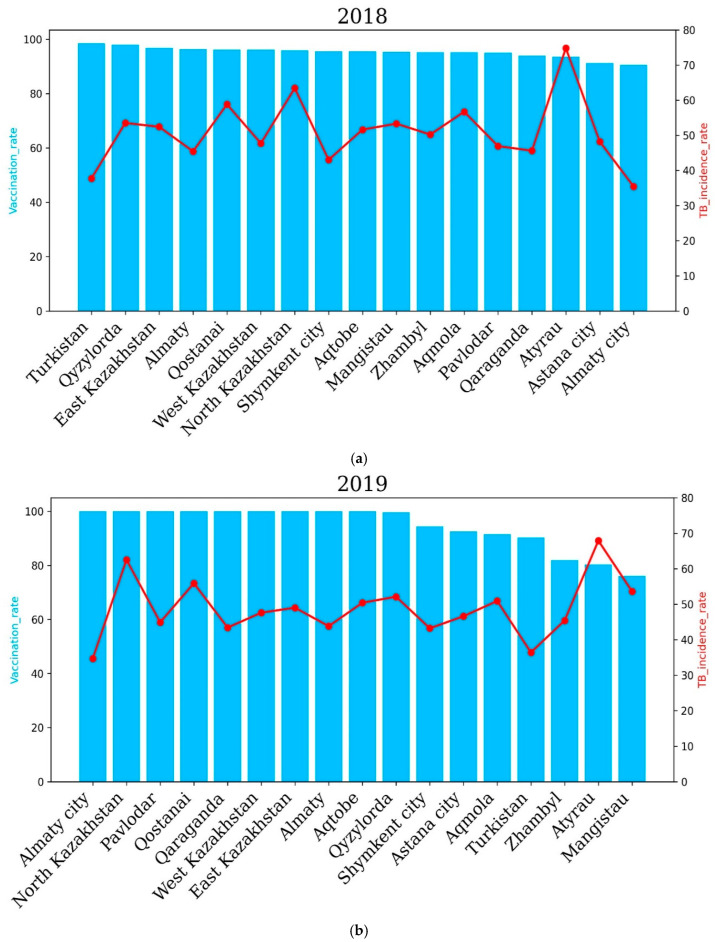
Regional tuberculosis (TB) incidence rates and vaccination coverage across Kazakhstan before COVID-19 for the years 2018 (**a**) and 2019 (**b**). Blue bars represent regional vaccination coverage rates (%), while red lines indicate TB incidence rates (cases per 100,000 population). The upper panel shows the data for 2018, and the lower panel illustrates the situation in 2019. In 2020, the Qyzylorda, Atyrau and Mangistau regions recorded a higher incidence rate of tuberculosis compared to Turkestan, Akmola, Shymkent, East Kazakhstan, Zhambyl and other oblasts. In 2020, incidence rates of 27.22, 25.29 and 27.46 cases per 100,000 people were recorded in the Turkestan region, Shymkent city and Almaty city, respectively. Accordingly, the vaccination rate in these regions was 96.6%, 95.6% and 87.9%. In the Qyzylorda, Mangistau and Atyrau regions, the TB incidence rate amounted to 49.7, 48.71 and 56.92 cases per 100,000 people, respectively. Interestingly, the vaccination rate was high and amounted to 96.8% for the Qyzylorda region, 95.3% for the Mangistau region and 86.5% for the Atyrau region.

To assess the correlation between the level of vaccination of newborns and the prevalence of TB in Kazakhstan, we analyzed the past six years. Our results showed that in 2018, vaccination rates had an overall positive impact on TB incidence rates in some regions of Kazakhstan. For example, regions such as Turkestan, Almaty and a city of republican significance, Shymkent, with a higher percentage of vaccinated newborns showed lower TB incidence rates compared to the Atyrau and North Kazakhstan regions. The highest incidence rate of 74.8 cases per 100,000 people was recorded in Atyrau, although the vaccination rate was 93.5% The lowest incidence rate of tuberculosis in Turkestan (in 2018) was 37.7 cases per 100,000 and correlates with the vaccination rate, as in this region, it amounted to 98.5% (Figure 5).

In 2019, North Kazakhstan and Atyrau recorded the highest TB incidence rates, while Almaty city and the Turkestan region recorded the lowest TB incidence rates. The level of the tuberculosis incidence rate of the population in the country for 2019 compared to 2018 decreased by 4.17%, and 8436 newly detected cases of tuberculosis were registered (2018—8804). The incidence rate per 100,000 population amounted to 46.2 cases per 100,000 against 48.2 cases per 100,000 for 2018 (Figure 5).

The incidence rate of tuberculosis in the republic for 2020, compared to 2019, decreased by 20.7%, and 6694 newly detected cases of tuberculosis were registered (2019—8436). The incidence rate per 100,000 population amounted to 36.6 cases against 46.2 for 2019. The level of tuberculosis morbidity in the republic for 2021 compared to 2020 increased by 0.6%, and 6824 first-time TB cases were registered (2020—6694 cases). In 2021, the highest incidence of tuberculosis was observed in the Qyzylorda, Atyrau, Mangistau, West Kazakhstan and North Kazakhstan regions. The Qyzylorda and Atyrau oblasts recorded 46.1% and 62.9% TB incidence, respectively. Interestingly, the Qyzylorda and Atyrau oblasts were among the regions with the highest vaccination rates, at 97.1 percent for Qyzylorda and 91.2 percent for Atyrau, which correlates with the vaccination rates of newborns. The lowest incidence rates of 28, 28.7 and 23.1 cases per 100 thousand people were demonstrated by the Turkestan region and Almaty and Shymkent cities, respectively. Vaccination rates for the Turkestan region and Almaty and Shymkent cities were 98.5%, 87.2% and 94.8%, respectively. These results show that vaccination rates are positively correlated with TB incidence rates.

In 2022, the East Kazakhstan, Atyrau, Qyzylorda, West Kazakhstan, Qostanai and Almaty regions showed higher incidence rates compared to other regions of the country, which correlated with the level of vaccination of newborns. The East Kazakhstan and Atyrau regions demonstrated the highest TB incidence rates in 2022—61.64 and 59.58 cases per 100,000 people, respectively. Interestingly, the vaccination rate was 97 and 93.2 percent for the East Kazakhstan and Atyrau regions, respectively.

The Abai and Zhetysu regions showed the lowest TB incidence rates in 2022, with 18.3 and 16.99 cases per 100, respectively. Vaccination rates for the Abai and Zhetysu oblasts were 95.5% and 93.9%, respectively.

**Figure 6:**
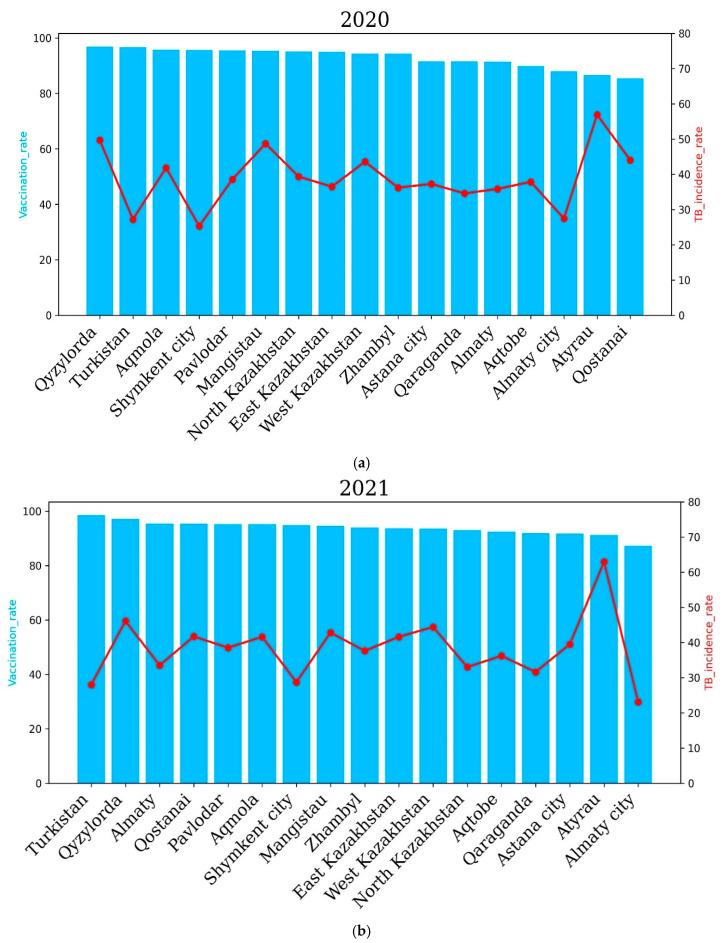

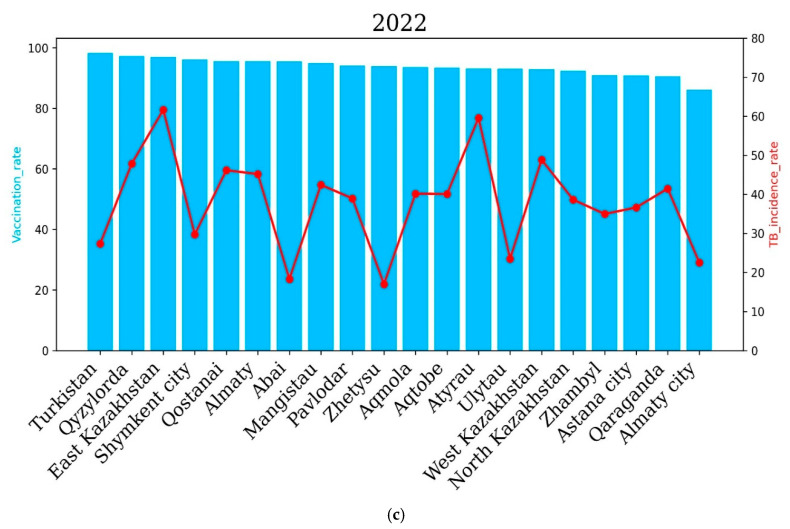
Regional tuberculosis (TB) incidence rates and vaccination coverage across Kazakhstan during the years 2020 to 2022. Blue bars indicate regional vaccination coverage rates (%), and red lines represent TB incidence rates (cases per 100,000 population). The upper panel shows data for 2020 (**a**), the middle panel for 2021 (**b**) and the lower panel for 2022 (**c**). The level of tuberculosis morbidity in the republic in 2020, compared to 2019, decreased by 20.7%, and 6694 first-time TB cases were registered (2019—8436). The incidence rate per 100 thousand population amounted to 36.6 against 46.2 in 2019. The level of tuberculosis morbidity in the republic for 2021 compared to 2020 increased by 0.6%, and 6824 first-time tuberculosis cases were registered (2020—6694 cases). In 2021, the highest incidence of tuberculosis was observed in the Qyzylorda, Atyrau, Mangistau, West Kazakhstan and North Kazakhstan regions. In the Qyzylorda and Atyrau regions, tuberculosis incidence rates of 46.1 and 62.9 cases per 100 thousand people were recorded, respectively. Interestingly, the Qyzylorda and Atyrau regions were among the regions with the highest vaccination rates, at 97.1 for Qyzylorda and 91.2 for the Atyrau region, which correlates with the level of vaccination of newborns. The lowest incidence rates were 28, 28.7 and 23.1 cases per 100,000 people in the Turkestan region, Almaty and Shymkent, respectively. Vaccination rates for the Turkestan region and Almaty and Shymkent cities were 98.5, 87.2 and 94.8 cases per 100,000 people, respectively. These results show that the vaccination rate is positively correlated with TB incidence.

The level of tuberculosis incidence in the country in 2022 compared to 2021 increased by 2.7%, and 7161 newly detected cases of tuberculosis were registered (6824 cases in 2021) (Figure 6).

In 2022, the East Kazakhstan, Atyrau, Qyzylorda, West Kazakhstan, Qostanai and Almaty regions demonstrated higher incidence rates compared to other regions of the country, which correlated with the level of newborn vaccination. The East Kazakhstan and Atyrau regions demonstrated the highest incidence rates of tuberculosis in 2022—61.64 and 59.58 cases per 100 thousand people, respectively. Interestingly, the vaccination rate was 97% and 93.2% for the East Kazakhstan and Atyrau regions, respectively. The Abai and Zhetysu regions demonstrated the lowest incidence rates of tuberculosis in 2022—18.3 and 16.99 cases per 100,000 people, respectively. The vaccination rate for the Abai and Zhetysu regions was 95.5% and 93.9%, respectively.

The incidence rate of tuberculosis in the population of the republic in 2022 compared to 2021 increased by 2.7%, and 7161 newly diagnosed cases of tuberculosis were registered (6824 cases in 2021).

**Figure 7:**
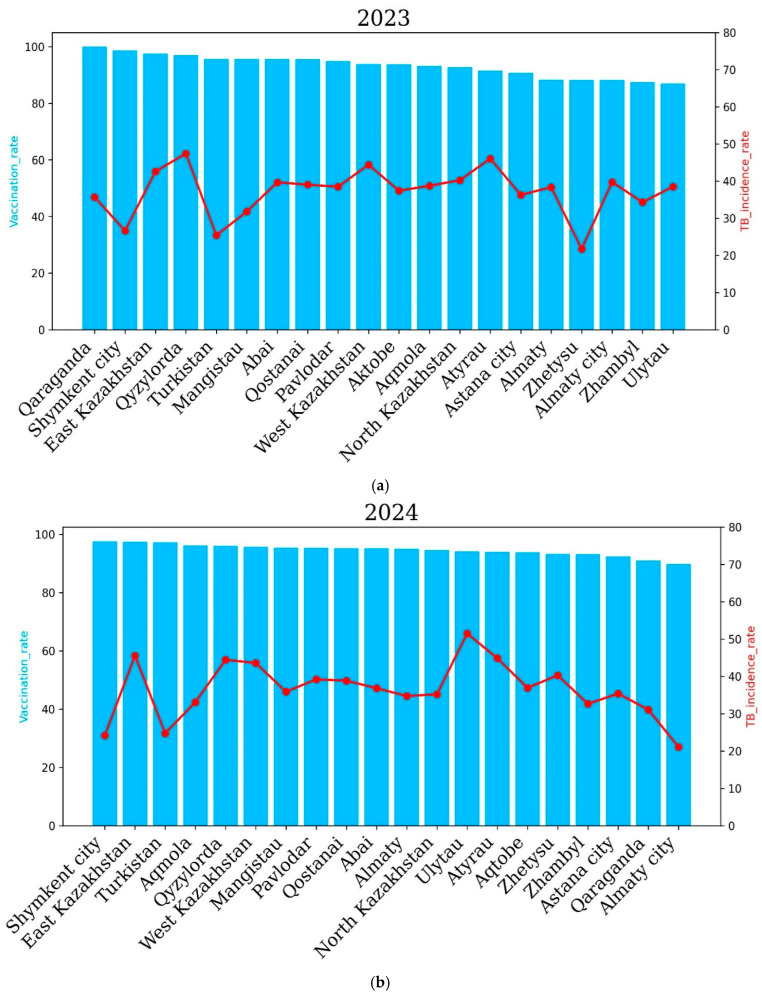
Regional tuberculosis (TB) incidence rates and vaccination coverage across Kazakhstan after COVID-19 for the years 2023 (**a**) and 2024 (**b**). Blue bars represent the vaccination coverage rates (%), and the red lines show the TB incidence rates (cases per 100,000 population). The top panel illustrates data from 2023, while the bottom panel presents data from 2024.

In 2023, a higher incidence rate of tuberculosis was noted in the Qyzylorda, Qaraganda, East Kazakhstan, West Kazakhstan and Atyrau regions. The Qyzylorda and Atyrau regions demonstrated the highest incidence rates—47.42 and 46.04 cases per 100,000 people, respectively. However, the vaccination rate was high in these regions. It is noteworthy that in 2023, no region recorded a tuberculosis incidence rate of more than 50 cases per 100 thousand people, which indicates an improvement in the situation of the spread of tuberculosis in the country as a whole. It is important to note that the overall incidence of tuberculosis significantly decreased compared to 2022, which demonstrated a higher level of vaccination of newborns and an improvement in the situation of the spread of tuberculosis in the country as a whole. In 2024, a higher incidence of tuberculosis was noted in the East Kazakhstan, Qyzylorda, West Kazakhstan, Ulytau, Atyrau and Zhetysu regions compared to other regions of Kazakhstan. The highest incidence of tuberculosis was recorded in Ulytau. The incidence of tuberculosis was 51.49 cases per 100,000 people in Ulytau, and the vaccination rate was 94.1%. The lowest incidence of tuberculosis was demonstrated by the cities of Almaty and Shymkent and the Turkestan region—21.1, 24.2 and 24.7 percent, respectively. It is important to note that in 2024, the vaccination rate was at least 90 percent in all regions and cities of the country. It is noteworthy that in 2024, an improvement in the incidence of tuberculosis was also observed in the country as a whole, since in no region did the incidence of tuberculosis exceed 50% (Figure 7).

To assess the association between age and TB prevalence, we analyzed the incidence of TB among different age groups including children, adolescents, adults and the general population for the past 6 years from 2018 to 2024. During 2018–2019, TB incidence rates were 6.8 and 6.6 cases per 100,000, respectively, among children. Adolescents demonstrated 47.9 and 46.7 cases per 100,000 people while adults demonstrated 65.2 and 62.58 cases per 100,000 people for tuberculosis incidence during 2018–2019. The general population demonstrated 48.2 and 46.16 cases per 100,000 people during the years 2018–2019. The TB incidence rate was 5.5, 5.2 and 5.13 cases per 100,000 people during 2020–2023, respectively, among children. Adolescents showed 30.3, 30.8 and 30.46 cases per 100,000 people while adults showed 50.3, 49.1 and 51.03 cases per 100,000 people for TB incidence during 2020–2023. During 2023–2024, TB incidence was 3.3 and 5.5 cases per 100,000 people, respectively, among children. Adolescents showed 25.39 and 26.4 cases per 100,000 people while adults showed 65.64 and 45.26 cases per 100,000 people for TB incidence during 2023–2024. These results underscore the need for comprehensive public health strategies that go beyond vaccination to achieve sustained reductions in TB incidence.

**Figure 8:**
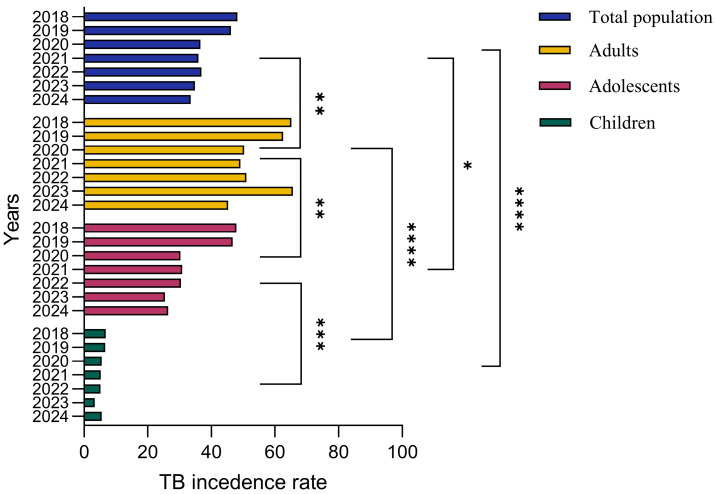
TB incidence rates by age group in Kazakhstan in 2018-2024. The bar graph illustrates annual TB incidence rates (cases per 100,000 population) segmented into total population, adults, adolescents and children. Adults consistently exhibit the highest incidence rates, followed by adolescents, while children demonstrate the lowest incidence. Over the observed period, incidence rates across all demographic groups generally show a declining trend, reflecting improvements in TB management and targeted intervention programs. The data were analyzed by a One-way ANOVA test with a Tukey test, and the *p* value indicates a significant difference. The asterisks (*, **, ***, ****) represent levels of statistical significance for differences in TB incidence rates between population groups and across years, where * indicates significant, ** indicates highly significant, *** indicates very highly significant, and **** indicates extremely significant differences.

Overall, there is a steady downward trend in tuberculosis incidence among adults and adolescents, with marked year-to-year variation, while children have the lowest incidence rates. Tuberculosis incidence among adults reached a peak around 2018–2019 and decreased slightly in the following years, while the adolescent group demonstrated a relatively stable trend. The overall incidence of tuberculosis in the population reflects cumulative trends across all groups, while the incidence among children remains significantly lower (Figure 8).

## 4. Discussion

This study aimed to identify the impact of the COVID-19 pandemic and socioeconomic factors (unemployment rate) on incidence and mortality over the past seven years (2018–2024). The period 2018–2022 had the highest incidence and mortality, potentially exacerbated by the COVID-19 pandemic, also shown in other countries [23], and entailed a number of other factors including economic recession [24,25] over the seven years studied. In Kazakhstan, the number of deaths (per 100,000 people) from tuberculosis decreased by 5.7% from 2022 to 2023, while globally, it decreased from 1.32 million people in 2022 to 1.25 million in 2023, a decrease of about 5.3%, indicating effective control measures in the country [6,8]. In 2024, tuberculosis incidence (per 100,000 people) and mortality were significantly reduced by 30.5% (to 33.5) and 58.3% (1.0), respectively, reflecting improved health outcomes in the post-pandemic period.

It is noteworthy that most western regions are characterized by high and medium unemployment rates and high incidence [26], which could be due to socioeconomic factors in these regions, which is also emphasized in the works [26,27]. At the same time, the western regions prevailed in terms of incidence throughout time, while in the northern regions, the incidence decreased by the post-pandemic period. At the same time, the western regions had relatively low mortality, indicating effective treatment methods but little prevention of the spread of the disease itself [27]. Northern regions, which were not inferior to western regions in terms of morbidity, are characterized by a colder, sharply continental climate, with the prevalence of winter, which facilitated the transmission of tuberculosis and seasonal fluctuations in immunity [28,29]. Generally, the northern, central, southeastern and eastern regions had low incidence during the COVID-19 period, especially in 2021. In these regions, the incidence was consistently low, especially in Almaty, Turkestan, Zhambyl and Shymkent, possibly due to the milder climate preventing a decrease in the overall health of the country (Figure 1 and Figure 2).

In addition, attention should be paid to the megacities of Almaty and Shymkent, which were characterized by high unemployment but maintained a low incidence, possibly due to the quality of medical care. Regions with the highest unemployment rates (Figure 3), such as Almaty and Turkestan, reported relatively low TB incidence, while regions with lower unemployment rates, such as Ulytau, continued to show elevated TB incidence rates. Although socioeconomic factors (Figure 3 and Figure 4), especially unemployment, were hypothesized to be strongly associated with TB incidence, the results indicate that unemployment rates alone cannot fully explain the burden of the disease [30]. It is important to note that Blas and Sivasankara emphasize the significant influence of the socioeconomic status of a region on the prevalence of TB cases. In addition, the growth of urbanization has a positive effect on reductions in morbidity, which was revealed in a comprehensive analysis of regional and socioeconomic factors using multiple linear regression (MLR) analysis. At the same time, the impact of unemployment on TB morbidity had, according to statistical analysis, the opposite effect, where the growth of unemployment tended to reduce morbidity and was not significant as a factor. As a result of the MLR analysis, the regional factor showed a characteristic effect on morbidity, which may indicate the quality of medical care, standard of living and other factors.

Vaccination plays an important role in controlling tuberculosis spread, though its impact varies. Our results demonstrated that the Turkistan region and Almaty city, with high vaccination rates, showed lower TB spread over all 6 years, suggesting a positive association between vaccination and reduced transmission. However, Atyrau, Qyzylorda and Qostanai, despite high vaccination coverage, demonstrated high TB spread. This indicates that other factors, such as socioeconomic conditions, environmental influences and healthcare accessibility, also play critical roles in TB transmission dynamics [31]. These findings underscore the limitations of using vaccination rates as a sole proxy for population-level protection. Vaccination coverage data may not fully capture the actual immunity within the population due to factors such as variations in vaccine effectiveness, waning immunity over time or potential inaccuracies in vaccination reporting. Moreover, the effectiveness of vaccination in preventing TB spread is influenced by the effective contact rate and social determinants of health, including living conditions and mobility patterns [32].

It has been known that age-related differences in disease risk are accompanied by differences in the response to infection and clinical features of disease, since primary infection with *Mycobacterium tuberculosis* is greatest in infants (younger than 4 years) and declines slowly to a nadir at 5–10 years of age. During adolescence (15–19 years of age), there is a rapid increase in risk with a second peak between the ages of 20 and 30 years [33]. Understanding the trends in TB between different age groups has crucial importance; thus, in our study, we analyzed three different age groups including total population. Our results demonstrated that the lowest TB incidence was in children, followed by adolescents, during the past 6 years. Adults demonstrated the highest TB incidence, which might be associated with lower immune system function at older ages [34,35]. Importantly, the spread of TB among the total population improved significantly in the past four years compared to between the years 2018 and 2019, suggesting enhanced public health interventions and TB control measures.

This study describes the unfavorable epidemic situation of tuberculosis in different regions of the Republic of Kazakhstan. It identifies regions with a high epidemiological burden of tuberculosis among the population. However, regions with consistently high incidence rates, such as Atyrau and Qyzylorda, require constant attention to prevent the spread of TB and demonstrate the need for targeted public health and social interventions. An analysis of the disease incidence for the years under study shows that a high percentage of the vulnerable population is the non-working population. Moreover, a higher vaccination rate is not always associated with lower TB incidence, as shown in the past seven years. These factors indicate a multifactorial influence on TB incidence. The COVID-19 pandemic had a profound impact on TB epidemiology in Kazakhstan, primarily through disruptions to healthcare services and public health programs [36]. Lockdowns and movement restrictions limited access to diagnostic facilities, resulting in delayed case detection and the under-reporting of TB cases [37]. Notably, an extremely high public health burden was associated with the registration of a large number of COVID-19 cases in the country, as COVID-19 cases were most likely prioritized rather than TB cases.

Building on these findings, future efforts should strengthen ongoing TB control by addressing the underlying social determinants of health. Research should focus on collecting individual-level data and conducting longitudinal studies to clarify causal relationships between socioeconomic factors, healthcare access and TB outcomes. Special attention is needed for high-incidence regions such as Atyrau and Qyzylorda through targeted public health interventions and improved healthcare services. Expanding vaccination outreach to vulnerable groups and integrating environmental factors into surveillance systems will further enhance disease prevention. Finally, fostering stronger coordination between government bodies, healthcare institutions and community organizations is essential to sustain progress and achieve long-term TB elimination. The findings of this study, while specific to Kazakhstan, have broader relevance for other countries with similar socioeconomic challenges and healthcare system constraints. The identified associations between unemployment, regional disparities, vaccination coverage, and TB incidence highlight the critical role of social determinants in TB transmission and control. These insights support the need for integrated TB control policies that combine medical interventions with socioeconomic development strategies. For Kazakhstan and comparable settings, adopting a multi-sectoral approach that strengthens healthcare infrastructure, reduces economic vulnerability and improves living conditions will be key to achieving sustainable TB control and meeting international public health targets. Despite providing valuable insights into the epidemiological trends in tuberculosis in Kazakhstan, this study has several important limitations. First, the analysis relied on aggregated regional data rather than individual-level data, which restricts the ability to control for critical confounding variables such as socioeconomic status, comorbidities, access to healthcare services and vaccination history. This limitation introduces the potential for ecological fallacy, where associations identified at the population level may not accurately reflect relationships at the individual level. Second, the observational and retrospective nature of this study limits the ability to establish causality between the examined factors—such as unemployment rates, vaccination coverage and climatic conditions—and tuberculosis incidence and mortality. While significant associations were observed, it is not possible to infer direct cause-and-effect relationships from the available data. Future research incorporating longitudinal individual-level data and advanced modeling techniques is necessary to validate these findings and better understand the causal mechanisms underlying tuberculosis transmission and outcomes.

## Figures and Tables

**Table 1:** Multiple linear regression model predicting TB incidence rate based on regional socioeconomic factors in Kazakhstan during 2018–2024.

Parameter Estimates	Variable	Estimate	Standard Error	95% CI (Asymptotic)	|t|	*p* Value	*p* Value Summary
β0	Intercept	102.5	32.31	38.20 to 166.7	3.172	0.0021	**
β1	B	−7.462	6.137	−19.67 to 4.747	1.216	0.2275	ns
β2	C	−1.626	0.3191	−2.261 to −0.9916	5.097	<0.0001	****
β3	D[Abai]	64.02	13.21	37.74 to 90.30	4.847	<0.0001	****
β4	D[Aqmola]	59.58	9.956	39.77 to 79.38	5.984	<0.0001	****
β5	D[Aqtobe]	93.78	16.70	60.55 to 127.0	5.614	<0.0001	****
β6	D[Almaty]	4.304	4.158	−3.968 to 12.58	1.035	0.3037	ns
β7	D[Atyrau]	81.42	10.98	59.58 to 103.3	7.418	<0.0001	****
β8	D[West Kazakhstan]	67.87	10.87	46.24 to 89.50	6.241	<0.0001	****
β9	D[Zhambyl]	39.72	7.151	25.50 to 53.95	5.555	<0.0001	****
β10	D[Zhetysu]	38.93	8.579	21.86 to 56.00	4.538	<0.0001	****
β11	D[Qaraganda]	98.65	18.99	60.86 to 136.4	5.194	<0.0001	****
β12	D[Qostanai]	74.98	12.22	50.67 to 99.29	6.136	<0.0001	****
β13	D[Qyzylorda]	56.63	8.338	40.04 to 73.21	6.791	<0.0001	****
β14	D[Mangistau]	47.18	7.443	32.37 to 61.98	6.339	<0.0001	****
β15	D[Pavlodar]	88.84	15.88	57.25 to 120.4	5.595	<0.0001	****
β16	D[North Kazakhstan]	54.98	8.756	37.56 to 72.39	6.279	<0.0001	****
β17	D[Ulytau]	97.43	19.29	59.07 to 135.8	5.052	<0.0001	****

## Data Availability

The dataset could be available on request.
